# mTOR regulates neuroprotective effect of immunized CD4+Foxp3+ T cells in optic nerve ischemia

**DOI:** 10.1038/srep37805

**Published:** 2016-11-25

**Authors:** Guochun Chen, Luosheng Tang, Wei Wei, Zhuo Li, Yunping Li, Xuanchu Duan, Huihui Chen

**Affiliations:** 1 Department of Nephrology, the Second Xiangya Hospital, Central South University, Changsha, Hunan, PR China; 2 Department of Ophthalmology, the Second Xiangya Hospital, Central South University, Changsha, Hunan, PR China

## Abstract

The therapeutic potential of targeting CD4+Foxp3+ regulatory T cells (Tregs) remains controversial under the condition of neuroinflammation. This study aims to explore the neuroprotective role of Tregs in optic nerve ischemia (ONI) and evaluate the therapeutic strategy of Tregs transfer with a focus on targeting the mammalian target of rapamycin (mTOR) pathway. Intraocular pressure was transiently increased in adult C57BL/6 mice to induce ONI. Mucosal tolerance of myelin basic protein (MBP) markedly increased retinal ganglion cell (RGC) survival after ONI through enhanced Tregs suppression. mTOR inhibition significantly promoted the frequency of MBP-immunized Tregs *in vitro* with increased production of anti-inflammatory cytokines. Transient rapamycin treatment highly promoted the immunosuppressive capacity of Tregs and inhibited retinal inflammation in ONI animals. Intravenous infusion of MBP-immunized Tregs, instead of regular Tregs, beneficially modulated immune activities of host retinal CD11b+ cells and CD4+ effector T cells, leading to significant improvement of RGC survival. Importantly, rapamycin treatment further enhanced the neuroprotective effect of Tregs transfer. Taken together, these findings reveal a fine regulation of mTOR signaling on immunized Tregs after acute retinal injury. Adoptive transfer with targeting-mTOR strategy markedly improves neuronal recovery after ONI, supporting the therapeutic potentials of Tregs in acute and chronic neurological disorder.

Neurodegeneration following acute ischemia or traumatic injury is one of the most frequent causes of permanent disability worldwide^1^. Despite considerable advances in the understanding of the pathophysiology of ischemic neural injury, therapeutic strategies for the subsequent progression of neurodegeneration remain limited^2^. Cumulative studies have highlighted that autoimmune reactions against exposed antigens of central nervous system (CNS) paly a major role in the development of traumatic neurodegeneration^3^. Induction of a regulatory immune response to myelin basic protein (MBP) before cerebral ischemia injury can prevent deleterious autoimmune response and improve neurological outcome after stroke^4^. It has been known that autoimmune T cells that are specific for MBP can protect adjacent neurons from the catastrophic secondary degeneration after the traumatic damage of CNS^5^. Among them, the latest findings suggest that a subpopulation of lymphocytes with regulatory effects is expected to beneficially regulate the neural antigen-induced interactions between neurons and immune cells.

Naturally occurring CD4+CD25+ regulatory T lymphocytes (Tregs), defined by expression of the transcription factor forkhead box P3 (Foxp3), are a developmentally and functionally distinct T cell subpopulation, which is indispensable for the regulation of immune response to self-antigens^6^. The majority of natural Foxp3+ Tregs are produced by the thymus as an antigen-primed T cell subpopulation specialized for immune suppression while a portion of them could differentiate from naïve conventional T cells (Tconvs) in the periphery under certain conditions^7^,^8^. Tregs are capable of modulating other immune cells via direct interaction and/or secretion of anti-inflammatory molecules, such as IL-10 and TGF-β1^9^. Immune dysregulation, characterized by constitutional and/or functional abnormalities in Tregs has been widely reported in the pathogeneses of various neuroinflammatory diseases^10^. On the contrary, prophylactic induction of Tregs response is associated with improved outcome after CNS ischemia^4^. These findings support a protective role of Tregs against pathogenic neuroinflammation. However, it is worth noting that natural Tregs are not a homogeneous population accompanying the process of neuroinflammation and can be further differentiated into distinct subsets with different functional features^11^. The molecular mechanisms that maintain the suppressive phenotype of Tregs in neuroimmunological events have yet to be clarified, resulting in the delay of further clinical practice.

The mammalian target of rapamycin (mTOR) is an evolutionarily conserved serine-threonine protein kinase that couples cellular activation to the environmental and intracellular nutritional status^12^. The most studied readouts of mTOR function are the p70S6 kinase (p70S6K) and the S6 ribosomal protein (S6), which are direct downstream translational regulators. Functioning in at least two distinct complexes (mTORC1 and mTORC2), mTOR acts as a coordinator of signaling pathways that shape the immune response of T cells to various stimuli^13^. mTORC1 is sensitive to inhibition by rapamycin, which is currently known to differently affect the function and homeostasis of Tregs and Tconvs^14^. In a time- and dose-dependent manner, T-cell stimulation in the presence of rapamycin promotes Tregs frequency and acquisition of a regulatory phenotype^13^,^15^, suggesting mTOR inhibition favors the Tregs suppression and the conversion of peripheral Tconvs into Tregs. However, the mechanism of mTOR regulation in Tregs activity remains unclear under the condition of neuroinflammation, such as acute retinal ischemia. It is necessary to fill in the gaps between mTOR regulation and Tregs-mediated neuroprotection in acute and chronic neurological disease.

In this study, we aim to explore the role of Tregs in neuroprotective immunity after acute retinal injury and determine the therapeutic strategy of Tregs transfer with a focus on targeting mTOR pathway. Firstly, we studied whether MBP-tolerance could improve RGC survival after optic nerve ischemia (ONI) and how it might impact the immune activity of Tregs. Secondly, we investigated the regulatory mechanism of mTOR pathway in the activation of immunized Tregs. Lastly, we studied the impact of Tregs transfer on host immune cells after acute retinal injury and evaluated the therapeutic potential of adoptive transfer with targeting-mTOR manipulation. Our findings may open a new way to the understanding of Tregs immunity in acute and chronic neurological disorders.

## Results

### Neural antigen-tolerance ameliorates retinal injury after ONI

**Figure 1:**
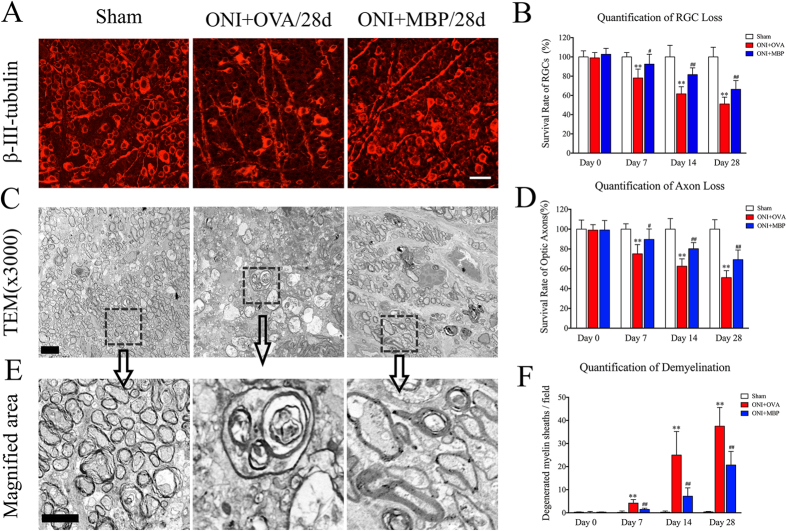
Neural antigen-immunization ameliorates retinal ischemic injury. Mucosal MBP-immunization was performed in adult C57BL/6 J mouse as described in Methods. Retina samples were harvested at day 0, 7, 14, and 28 of reperfusion. (**A**) Representative image of retinal flat mount with an antibody against β-III-tubulin. Scale bar = 10 μm; (**B**) Quantitative analysis of survival RGCs based on retinal flat mount; (**C**) Representative TEM image of mouse optic nerve cross-sections; (**D**) Quantitative analysis of survival axons based on TEM detection, Scale bar = 5 μm; (**E**) Magnified image of square areas in (**C**), Scale bar = 2.5 μm; (**F**) Quantitative analysis of demyelinated axons based on the TEM detection. All experiments were performed in triplicate. Data are presented as mean ± SD; n = 6–8 animals in each group; **P < 0.01, ONI + OVA vs. sham group; ^#^P < 0.05, ^##^P < 0.01, ONI + MBP vs. ONI + OVA group. TEM, Transmission electron microscopy; ONI, optic nerve ischemia; OVA, ovalbumin; MBP, myelin basic protein; RGC, retinal ganglion cell.

A protective role of MBP tolerance has been cumulatively reported in experimental models of autoimmune neurological disorder^16^ and stroke^4^,^17^. However, the effect of MBP tolerance on acute retinal injury remains unknown. In comparison with sham animals, ischemic retina suffered from increasing RGC loss with time, which was significantly ameliorated by immunologic tolerance to MBP (Fig. 1A,B). Apart from improvement of RGC survival, the MBP-immunized animals exhibited better axon protection than the OVA group. TEM detection on mouse optic nerve revealed that transient ischemia induced significant axonal damage, which was markedly ameliorated in MBP-immunized animals at each follow-up time point (Fig. 1C–F). Specifically, severe axon loss, including depletion of both large and small-diameter axons, became apparent with time after ONI. Demyelinated axons with nearby accumulated myelin breakdown debris were scattered in regions with low axon-packing density. A mass of axons became atrophy with the axolemma separated from the innermost layer of myelin lamellar membrane while the myelin sheath degenerated with a vacuolated or reticulated appearance. However, optic nerve from the eye with MBP-immunization showed prominent neuronal protection, indicated by more well-myelinated axons and less neuronal degeneration (Fig. 1C,E). Further quantification analysis of axon loss (Fig. 1D) and demyelination (Fig. 1F) confirmed the protective effect of MBP tolerance on ischemic retina.

### Mucosal tolerance of MBP modulates Tregs activity in acute retinal injury

**Figure 2:**
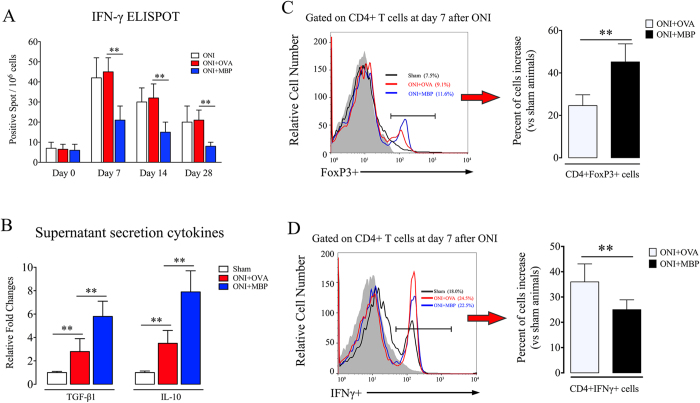
Neural antigen-immunization modifies immune activity of CD4+ T cells after ONI. Animal experiments were performed as described in Methods. For ELISPOT and ELISA assays, freshly isolated mouse splenocytes were cultured in media with MBP (25 μg/mL) for 48 hours and applied to the examinations. (**A**) ELISPOT assay of IFN-γ. (**B**) ELISA assay of TGF-β1 and IL-10 secretion in cell culture supernatants. (**C**) Flow cytometry assay of CD4+Foxp3+ T cells isolated from mice eye-draining lymph nodes at day 7 of reperfusion. (**D**) Flow cytometry assay of CD4+ IFNγ+ T cells isolated from mice eye-draining lymph nodes. All experiments were performed in triplicate. Data are presented as mean ± SD; *P < 0.05, **P < 0.01; n = 6–8 animals in each group. The gray histogram of flow cytometry represents the isotype-matched negative control. ONI, optic nerve ischemia; OVA, ovalbumin; MBP, myelin basic protein.

It has been revealed that antigen tolerance alters the activity of Tconvs (potentially damaging) and Tregs (potentially beneficial) in autoimmune neurological disease^18^,^19^. We next sought to determine whether and how MBP tolerance might influence CD4+ T cells after ONI. As indicated by ELISPOT, retinal injury highly promoted IFN-γ production in spleen immune cells, indicating a strong inflammatory response to ONI (Fig. 2A). However, secretion of IFN-γ was markedly reduced in immunized splenocytes. Meanwhile, ELISA assay on the culture supernatants showed that MBP tolerance further enhanced the secretion of IL-10 and TGF-β1 in immune cells, which had been initially triggered by retinal injury to limit intense inflammatory damage to self (Fig. 2B). CD4+ T cells consist of diverse subpopulations with distinct immune functions. Using flow cytometry, we found an increased frequency of both CD4+Foxp3+ T cells (Fig. 2C) and CD4+IFN-γ+ T cells (Fig. 2D) in mouse eye-draining lymph nodes after ONI, reflecting a complementary immune response to ONI. Importantly, MBP tolerance significantly increased the number of CD4+Foxp3+ T cells but decreased that of CD4+ IFN-γ+ T cells in mouse eye-draining lymph nodes.

**Figure 3:**
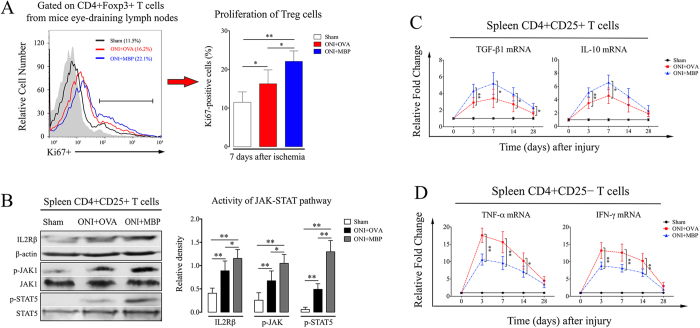
Neural antigen-immunization enhances Tregs proliferation and suppressive capacity after ONI. Animal experiments were performed as described in Methods. T cells were freshly isolated from mice spleens or eye-draining lymph nodes. (**A**) Flow cytometry assay of ki67 expression gated on CD4+Foxp3+ T cells at day 7 of reperfusion. (**B**) Western blot and relative density analysis on CD4+CD25+ T cells for activities of IL2Rβ-JAK-STAT5 pathways at day 7 of reperfusion. (**C**) realtime-PCR analysis on CD4+CD25+ T cells at each follow-up time point for mRNA expression of TGF-β1 and IL-10. (**D**) realtime-PCR analysis on CD4+CD25− T cells at each follow-up time point for mRNA expression of TNF-α and IFN-γ. All experiments were performed in triplicate. Data are presented as mean ± SD; *P < 0.05, **P < 0.01; n = 6–8 animals in each group. The gray histogram of flow cytometry represents the isotype-matched negative control. ONI, optic nerve ischemia; OVA, ovalbumin; MBP, myelin basic protein.

To further evaluate the regulation of MBP tolerance on Tregs activity, we sorted CD4+Foxp3+ T cells from mice eye-draining lymph nodes. About 11.5% of Tregs in sham animals were positive for ki67 expression, indicating a portion of Tregs normally maintained proliferation. Retinal ischemia highly induced an increase of Tregs proliferation, which was more prominent in MBP-immunized animals (Fig. 3A). IL-2Rβ-JAK-STAT5 pathway is fundamental for Tregs suppression^20^,^21^. We observed increased phosphorylation of STAT5 in CD4+CD25+ T cells (>90% pure by FACS analysis) after ONI, which was concomitant with enhanced expression of IL-2Rβ and p-JAK1. Activation of STAT5 pathways was further promoted in CD4+CD25+ T cells with MBP tolerance (Fig. 3B). As shown by realtime-PCR assay at each follow-up time point, mRNA expression of immunosuppressive cytokines, including TGF-β1 and IL-10, markedly increased in CD4+CD25+ T cells after retinal injury, which peaked at day 7 of reperfusion. The suppressive capacity of Tregs was further enhanced in MBP-immunized animals (Fig. 3C). Conversely, MBP tolerance dramatically reduced the mRNA expression of TNF-α and INF-γ in CD4+CD25− T cells (Fig. 3D), which had been considered as the major responder T cells in the progression of neural inflammation. Taken together, these data suggest that mucosal tolerance of neural antigen beneficially enhances Tregs immunosuppression in acute retinal injury.

### mTOR signaling regulates retinal Tregs immunosuppression

**Figure 4:**
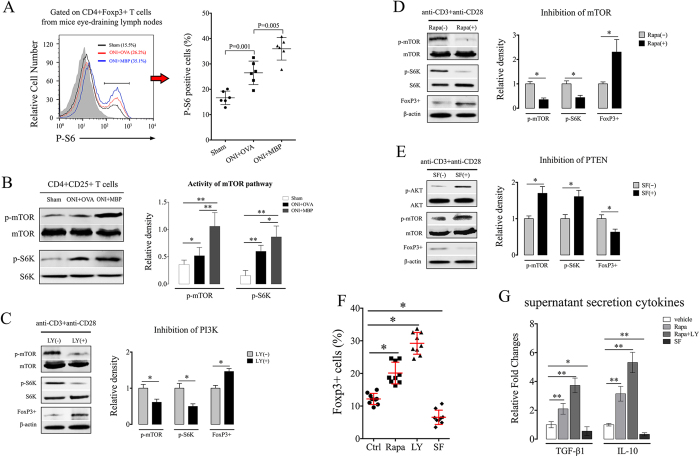
mTOR signaling regulates Tregs activity in neural antigen-immunized animals. Animal experiments were performed as described in Methods. *In vitro* experiments, CD4+ T cells were isolated from MBP-immunized animals at day 7 of reperfusion and treated with ly294002 (10 μM), rapamycin (25 nM), or SF1670 (500 nM) for 1 hour before co-stimulations with anti-CD3 and anti-CD28 for 36 hours. (**A**) Flow cytometry assay of P-S6 expression in CD4+Foxp3+ T cells at day 7 of reperfusion. (**B**) Western blot for phosphorylation of mTOR and S6K in spleen CD4+CD25+ T cells at day 7 of reperfusion. (**C–E**) Western blot for phosphorylation of mTOR pathway and the Foxp3 expression in culture CD4+ T cells with the presence of ly294002 (**C**), rapamycin (**D**), and SF1670 (**E**). (**F**) Flow cytometry assay of Foxp3 expression in culture CD4+ T cells. (**G**) ELISA assay on secretion of TGF-β1 and IL-10 in cell culture supernatants. All experiments were performed in triplicate. Data are presented as mean ± SD; *P < 0.05, **P < 0.01. ONI, optic nerve ischemia; OVA, ovalbumin; MBP, myelin basic protein. SF, SF1670; LY, ly294002; Rapa, rapamycin.

mTOR plays different roles in T cell immunity^13^,^14^,^22^. We next studied whether and how mTOR may regulate immune activity of immunized Tregs. By flow cytometry assay, we showed that baseline phosphorylation of S6 was evident in non-reactive CD4+Foxp3+ T cells from eye-draining lymph nodes. After ONI, P-S6 expression in Tregs was markedly enhanced, particularly prominent in MBP-immunized animals (Fig. 4A). Western blot assay on spleen CD4+CD25+ cells (>90% pure by FACS analysis) further confirmed the phosphorylation pattern of mTOR pathway, which was significantly higher in MBP-immunized animals than vehicle groups after ONI (Fig. 4B). For *in vitro* experiments, immunized CD4+ T cells were isolated from mouse spleen at day 7 of reperfusion. Transient inhibition of PI3K/mTOR pathway, with either PI3K inhibitor (ly294002) or mTOR inhibitor (rapamycin), resulted in a significant increase of Foxp3 expression in culture CD4+ T cells after Tregs specific co-stimulations (Fig. 4C,D). Conversely, SF1670, a small molecule inhibitor for phosphatase and tensin homologue (PTEN)^23^, markedly increased AKT/mTOR phosphorylation in CD4+ T cells with an inhibitory effect on their Foxp3 expression (Fig. 4E). Consistent with western blot assay, flow cytometry confirmed the regulatory effect of mTOR signaling on Tregs frequency of culture CD4+ T cells. Inhibition of mTOR pathways by rapamycin or ly294002 significantly expanded CD4+Foxp3+ T cells while activation of mTOR pathways by SF1670 decreased the percentage of Foxp3+ T cells (Fig. 4F). ELISA assay on cell culture supernatants showed that transient incubation of rapamycin and ly294002 before anti-CD3/CD28 stimulations markedly increased the production of suppressive cytokines in culture CD4+ T cells, including IL-10, and TGF-β1. On the contrary, inhibition of PTEN by SF1670 reduced these secreting cytokines in cell culture supernatants (Fig. 4G).

**Figure 5:**
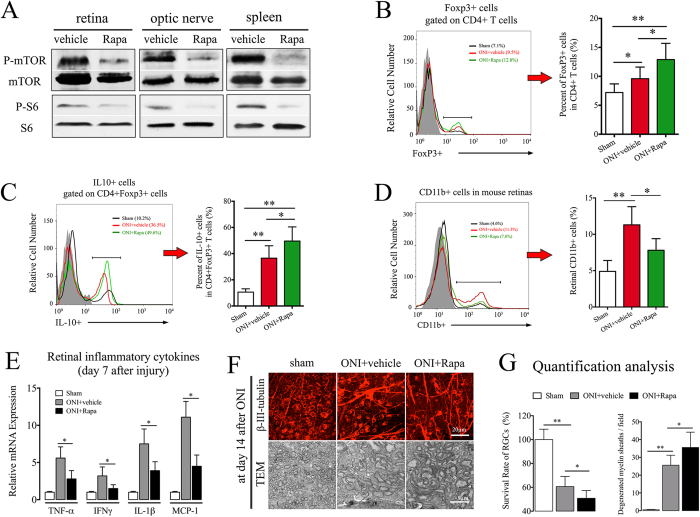
Rapamycin promotes Tregs activity after ONI. A single dose of rapamycin (2 mg/kg) was administered to animals 24-hour before the initiation of ONI. (**A**) Western blot probed for phosphorylation of mTOR pathways in mouse tissues of retina, optic nerve, and spleen at day 3 after ONI. (**B**) Flow cytometry assay of Foxp3 expression in CD4+ T cells in mouse eye-draining lymph nodes at day 7 after ONI. (**C**) Flow cytometry assay of IL-10+ expression in CD4+Foxp3+ T cells from mouse eye-draining lymph nodes at day 7 after ONI. (**D**) Flow cytometry assay of retinal CD11b+ cells at day 7 after ONI. (**E**) realtime-PCR analysis of inflammatory cytokines in mouse retina, including TNF-α, IFN-γ, IL-1β, and MCP-1. (**F**) Representative image of retinal flat mount and TEM detection of optic nerve cross-sections. (**G**) Quantitative assessment of RGC survival and axon demyelination at day 14 after ONI. All experiments were performed in triplicate. Data are presented as mean ± SD; *P < 0.05, **P < 0.01. n = 6–8 animals in each group. The gray histogram of flow cytometry represents the isotype-matched negative control. ONI, optic nerve ischemia; Rapa, rapamycin; TEM, Transmission electron microscopy; RGC, retinal ganglion cell.

To evaluate *in vivo* regulation of mTOR signaling on Tregs immunity, a single dose of rapamycin was administered to animals 24-hour prior to ONI. As indicated by western blot, systemic administration of rapamycin markedly inhibited mTOR activity in mouse retina, optic nerve, and spleen after ONI (Fig. 5A). According to flow cytometry assay, rapamycin induced a higher frequency of CD4+Foxp3+ Tregs in mouse eye-draining lymph node when compared to vehicle-treated animals (Fig. 5B), indicating that transient inhibition of mTOR signaling promoted Tregs expansion after neural injury. We next examined effect of rapamycin on the regulatory capacity of expanded Tregs. IL-10 is one of the best-characterized inhibitory cytokines produced by active Tregs^11^,^24^. Only a small proportion of CD4+Foxp3+ cells expressing IL-10 were detected in eye-draining lymph nodes of sham animals. However, the frequency of IL-10 expression markedly increased in CD4+Foxp3+ cells after ONI, suggesting retinal inflammation triggered the regulatory response of Tregs to limit self-damage. Importantly, rapamycin further promoted the increased frequency of IL-10 expressing Tregs (Fig. 5C). Given the suppressive regulation of active Tregs on other inflammatory cells^25^, these results thus indicate that rapamycin can partly exert its anti-inflammatory effect through enhancement of Tregs suppression.

It is worth noting that rapamycin also directly inhibits inflammatory activities of diverse immune cells, such as macrophages and dendritic cells^26^. In this study, we observed that rapamycin markedly reduced the numbers of infiltrating CD11b+ cells in injured retina (Fig. 5D) and ameliorated retinal inflammatory mRNA expression of TNF-α, IFN-γ, IL-1β, and MCP-1 (Fig. 5E). Despite its anti-inflammatory effect, rapamycin treatment did not contribute to neural recovery after ONI. On the contrary, retinal flat mount and TEM detection demonstrated that rapamycin aggravated RGC loss and axon demyelination at day 14 of reperfusion (Fig. 5F,G). Given a beneficial role of mTOR signaling in axon regeneration after nerve injury^27^,^28^, systemic inhibition of mTOR signaling could undermine the process of RGC self-recovery and axon regeneration.

### mTOR inhibition enhances the therapeutic effect of Tregs transfer on retinal injury

**Figure 6:**
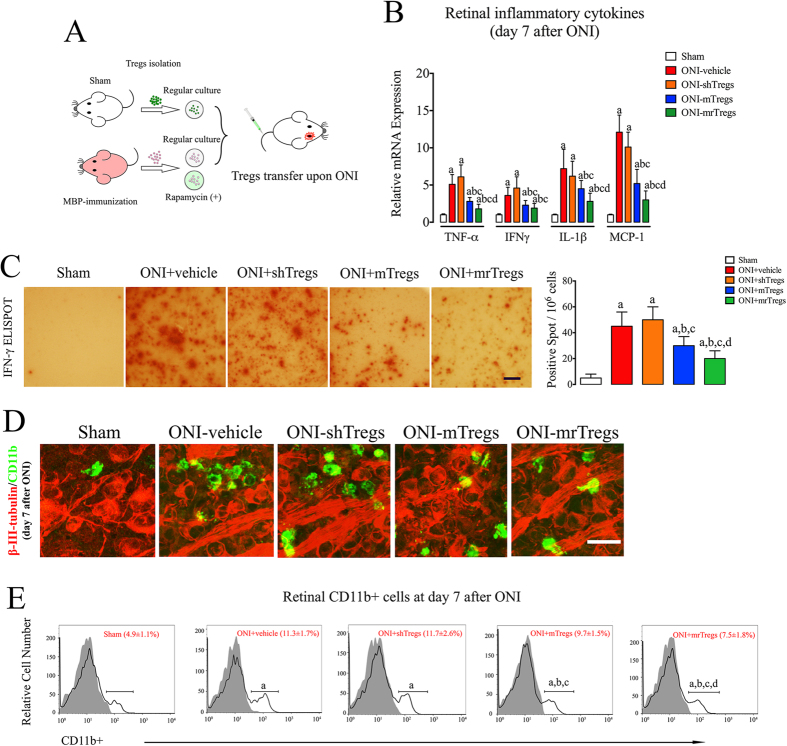
Rapamycin enhances immunoregulatory effect of Tregs transfer on retinal inflammation. (**A**) Schematic model of adoptive transfer. CD4+CD25 + T cells were isolated from spleens of sham or MBP-immunized mice (>90% pure by FACS analysis). Cells were then cultured for 1 h with or without the presence of rapamycin (25 nM) and applied to intravenous infusion upon ONI. (**B)** realtime-PCR analysis on mRNA expression of inflammatory cytokines in mouse retina, including TNF-α, IFN-γ, IL-1β, and MCP-1. **(C**) ELISPOT assay on IFN-γ secreting cells. Splenocytes were freshly isolated at day 7 after ONI and applied to the examinations. Scale bar = 1mm. (**D**) Representative image of retinal flat mount with antibodies against β-III-tubulin (red) and CD11b (green). Scale bar = 10 μm. (**E**) Flow cytometry assay of retinal CD11b+ cells at day 7 after ONI. The gray histogram of flow cytometry represents the isotype-matched negative control. All experiments were performed in triplicate. n = 6–8 animals in each group. Data are presented as mean ± SD; P < 0.05: a, vs. sham group; b, vs. ONI+ vehicle; c, vs. ONI+ shTregs; d, vs. ONI-mTregs. ONI, optic nerve ischemia; shTregs, Tregs from sham animals; mTregs, Tregs from MBP-immunized animals; mrTregs, Tregs from MBP-immunized animals with rapamycin incubation.

Adoptive therapy with Tregs represents a promising strategy in the clinical setting^29^. However, rising conflicts have been reported in recent studies that transfer of exogenous Tregs might exacerbate neuroinflammation after acute neural injury^30^,^31^. We next evaluated whether the manipulation strategy might influence the therapeutic effect of Tregs transfer on acute retinal injury. Adoptive transfer was performed upon ONI using sham Tregs (shTregs), MBP-immunized Tregs (mTregs), and MBP-immunized Tregs with rapamycin treatment (mrTregs) (Fig. 6A). As indicated by realtime-PCR assay at day 7 of reperfusion, shTregs did not reduce retinal mRNA expression of pro-inflammatory cytokines, including TNF-α, IFN-γ, IL-1β, and MCP-1. However, intravenous infusion of mTregs markedly suppressed the retinal inflammatory response to ONI. Importantly, rapamycin further enhanced the inhibitory effect of mTregs on retinal inflammation (Fig. 6B). ELISPOT assay confirmed the impact of Tregs transfer on host immune cells. Splenocytes from ONI animals were highly activated with abundant secretion of IFN-γ. mTregs, but not shTregs, markedly suppressed the production of IFN-γ in immune cells after ONI. The immunosuppression was particularly prominent in animals treated with mrT-tregs (Fig. 6C), suggesting that rapamycin markedly enhanced the suppressive capacity of mTregs after adoptive transfer.

Retinal CD11b+ microglial cells are the major inflammatory cells interacting with CD4+ effector T cells during retinal injury^32^,^33^. Immunofluorescent staining showed abundant CD11b+ cells surrounding the involved RGCs at day 7 of reperfusion (Fig. 6D). Although both of mTregs and mrTregs could markedly inhibited the activation of CD11b+ cells in injured retina, the latter strategy exerted a more prominent inhibition. The pattern of retinal CD11b+ cells was further confirmed by flow cytometry assay. A significant increase of CD11b+ cells was observed in mouse retina from sham (4.9%) to ONI animals (11.3%). No improvement in macrophage accumulation was achieved by infusion of shTregs (11.7%), which, however, was significantly reduced by adoptive transfer of mTregs (9.7%) and mrTregs (7.5%) (Fig. 6E). CD11b+ cells represent a heterogenous macrophage population in CNS with distinct functions of pro-inflammatory (M1) or anti-inflammatory (M2)^34^. It remains intriguing to explore the underlying interplays between Tregs and subtypes of CD11b+ cells after ONI.

**Figure 7:**
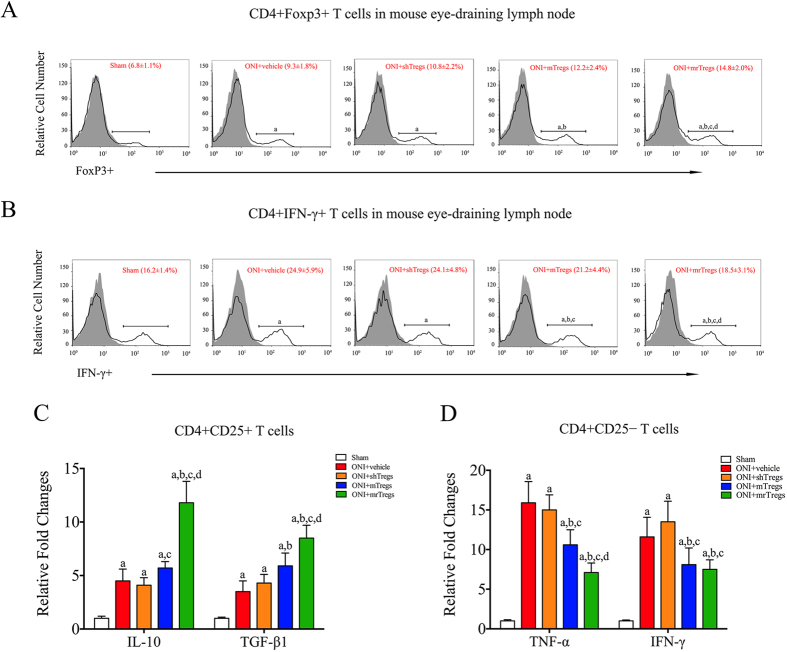
Transferred Tregs modulate immune activity of host CD4+ T cells after ONI. Animal experiments were performed as described in Fig. 6. Samples were harvested at day 7 after ONI and applied to next examinations. (**A**) Flow cytometry assay of CD4+Foxp3+ T cells from mice eye-draining lymph nodes. (**B**) Flow cytometry assay of CD4+ IFNγ+ T cells from mice eye-draining lymph nodes. (**C**) realtime-PCR analysis for mRNA expression of IL-10 and TGF-β1 in CD4+CD25+ T cells from mice eye-draining lymph nodes (>90% pure by FACS analysis). (**D**) realtime-PCR analysis for mRNA expression of TNF-α and IFN-γ in CD4+CD25− T cells from mice eye-draining lymph nodes (>90% pure by FACS analysis). All experiments were performed in triplicate. Data are presented as mean ± SD; n = 6–8 animals in each group. P < 0.05: a, vs. sham group; b, vs. ONI-vehicle; c, vs. ONI+shTregs; d, vs. ONI+mTregs. The gray histogram of flow cytometry represents the isotype-matched negative control. ONI, optic nerve ischemia; shTregs, Tregs from sham animals; mTregs, Tregs from MBP-immunized animals; mrTregs, Tregs from MBP-immunized animals with rapamycin incubation.

CD4+ T lymphocytes consist of diverse subpopulations with distinct immune functions^35^. After ONI, an increased frequency of both CD4+Foxp3+ T cells (from 6.8% to 9.3%) and CD4+IFN-γ+ T cells (from 16.2% to 24.9%) was observed in mouse eye-draining lymph nodes (Fig. 7A,B). A higher occurrence of CD4+Foxp3+ cells was shown after infusion of mTregs (12.2%), which was further promoted by mrTregs transfer (14.5%) (Fig. 7A). Conversely, the proportion of IFNγ+ in CD4+ T cells was significantly decreased by transfer of mTregs (21.2%), and particularly of mrTregs (18.5%) (Fig. 7B). realtime-PCR assay confirmed that mrTregs exerted higher immunosuppression than mTregs after adoptive transfer, leading to increased mRNA expression of IL-10 and TGF-β1 in host CD4+CD25+ T cells (Fig. 7C) but decreased mRNA expression of TNF-α and IFN-γ in host CD4+CD25− T cells (Fig. 7D). Compared to the vehicle treatment, no significant difference of immune response was found in CD4+ T cells after shTregs transfer.

**Figure 8:**
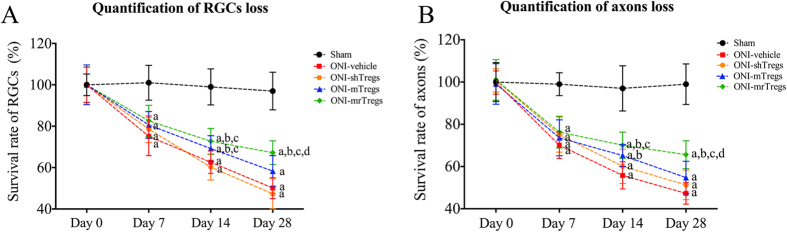
Tregs transfer improves neural recovery after ONI. Animal experiments were performed as described in Fig. 6. Samples were harvested at each follow-up time points after ONI and applied to next assays. (**A**) Quantitative assessment of RGCs loss based on mouse retinal flat mount. (**B**) Quantitative assessment of axons loss based on TEM assay of optic nerve cross-section. All experiments were performed in triplicate. Data are presented as mean ± SD; n = 6–8 animals in each group. P < 0.05: a, vs. sham group; b, vs. ONI-vehicle; c, vs. ONI-shTregs; d, vs. mTregs. ONI, optic nerve ischemia; shTregs, sham Tregs; mTregs, MBP-immunized Tregs animals; mrTregs, MBP-immunized Tregs with rapamycin incubation.

We lastly evaluated the therapeutic effect of Tregs transfer on the long-term outcome of neural injury. Retinal flat mount and TEM assay of optic nerve cross-sections were performed to assess the neuronal recovery. A progressive loss of RGC and axon was observed after acute retinal ischemia. Although no beneficial effect was achieved by shTregs, transfer of mTregs slowed down RGC loss (Fig. 8A) and axonal damage (Fig. 8B) at day 14 after ONI. Importantly, mrTreg significantly improved RGC survival at day 28 as well as day 14 after ischemia. Taken together, adoptive transfer of immunized Tregs, instead of regular Tregs, modifies immune response of host retinal macrophages and T cells after ONI. mTOR inhibition further enhances Tregs suppression, leading to significant neuronal protection on acute retinal injury.

## Discussion

In this study, we reveal a regulatory role of mTOR signaling in maintaining the functional Tregs against neuroinflammatory injury. We demonstrate that MBP-tolerance before ONI promotes Tregs activity, which beneficially ameliorates the retinal inflammation via abundant secretion of regulatory cytokines, leading to significant improvement in neuronal recovery from ONI (Figs 1, 2 and 3). Importantly, transient inhibition of mTOR signaling further enhances the suppressive capacity of Tregs (Figs 4 and 5). Adoptive transfer of immunized Tregs with mTOR inhibition beneficially modulates the host immune cells after ONI (Figs 6 and 7), contributing to a long-term therapeutic effect on retinal ischemic injury (Fig. 8). These findings provide novel insights into the underlying mechanisms of neural immune response to ONI, supporting the potential of targeting-mTOR strategy to strengthen regulatory capacity of Tregs in acute and chronic neurological disorder.

The regulatory mechanisms of Tregs can be classified into several major groups, including inhibitory cytokine release, cell-contacts dependent suppression, modulation of other suppressor cells, and metabolic disruption^36^. Although with different cell-tracking strategies of GFP transgenic animals and fluorescence dye labeling, we hardly observed migration of transfused Tregs into the injured retina (data not shown). However, the recruitment of host Tregs into ischemic retina is significant after adoptive transfer (Fig. 7A), which exert potent immunosuppression through abundant production of inhibitory cytokines (Fig. 7C). These findings thus suggest that transferred Tregs indirectly present their neural protection via host immune cells rather than direct contact with retinal resident cells. We suppose that exogenous Tregs may distribute in the circulatory system or the peripheral lymphoid organs after adoptive transfer. By direct cell-cell contacts in circulation or secretion of suppressor cytokines, these transferred Tregs suppress the effector T cells (CD4+ IFNγ+) and responder myeloid cells (CD11b+) but promote the activation of host regulatory T cells in inflammatory retina.

It is worth noting that Tregs are a heterogeneous population with a great magnitude of plasticity^37^,^38^. Recent studies suggest a variety of proinflammatory cytokines, such as IL-1, IL-6, and IL-12 are able to downregulate Foxp3 expression and convert Tregs into potentially pathogenic Tconvs under certain circumstances^39^. Due to their functional adaptation to various physiological situations, exogenous Tregs could differentiate into other cytokine-producing CD4+ T cells after adoptive transfer in the milieu of ongoing inflammation. We assume that Tregs, when transferred in the context of neuroinflammation, can potentially change their immunosuppressive phenotype to effector Tconvs under the influence of vast inflammatory cytokines. Therefore, the beneficial effect of Tregs transfer on tissue repair might be highly dependent on the time point considered. The phenotypic instability of exogenous Tregs may also explain the rising conflicting results in therapeutic Tregs transfers that both beneficial^18^,^40^,^41^ and detrimental^30^,^31^ effects can be observed on the host immune response to neuronal injury. One of the major findings in our study is that MBP-immunized Tregs, instead of regular Tregs, exert their protective immunity against neuroinflammation of ONI after adoptive transfer. We suppose that antigen-tolerance helps to stabilize the immunosuppressive phenotype of Tregs in a pro-inflammatory milieu after ONI. This notion is supported by previous findings that adoptive transfer of MBP-reactive Tregs, but not polyclonal (non-reactive) Tregs, can lead to efficient protection against neurodegeneration of CNS^18^. Importantly, the protective immunity of MBP-immunized Tregs can be further enhanced by mTOR inhibition. As mTOR inhibitors significantly promote the suppressive capacity of Tregs (Figs 4 and 5), we believe that the targeting-mTOR manipulation further enhances the phenotypic stability of transferred Tregs in host immune system. These results also highlight the importance of therapeutic strategy to strengthen the functional stability of Tregs before any application of adoptive transfer to neurological disorder.

Cumulative studies have increased our understandings on the molecular events involved in the fine regulation of Tregs^7^,^36^. Among them, mTOR signaling provides surprising insights into the nature of Tregs immunity^14^,^22^,^26^. In this study, it is intriguing to observe that either activation or inhibition of mTOR signaling correlates with Tregs response in acute retinal injury. Firstly, ki67 expression in Tregs correlates well with the phosphorylation of mTOR pathway after ONI (Figs 3A and 4A). In this context, mTOR activation might favor the Tregs self-proliferation/expansion, leading to an increase of Tregs in volume to regulate extensive inflammatory damage after ischemia. These data are in line with our knowledge that mTOR prominently controls most cell proliferation. Secondly, we have consistently demonstrated that mTOR inhibition can markedly promote the Tregs expansion and immunosuppression (Figs 4 and 5). In this context, Tregs expansion by mTOR inhibitors may be largely attributable to a phenomenon of increased “differentiation”, since mTOR inhibitors can markedly promote the conversion of conventional CD4+ T cells into CD4+Foxp3+ T cells^14^,^22^,^26^,^42^. Moreover, it is worth noting that several non-mTOR Foxp3-promoting signalings (e.g. IL-10 and TGF-β1) have been shown to be immediately upregulated at the onset of neuroinflammation, which may promote Tregs frequency and suppression in neural injury as well^9^. In agreement with recent findings, our study suggests that the role of mTOR signaling in Tregs biology is multifunctional and dynamic so that a “switch” in mTOR activation might be essential for the maintenance of Tregs activity^15^,^43^. Future investigations remain necessary to fully understand the complex mechanism of mTOR in Tregs activation.

As a multifunctional regulator, mTOR is active during neuron development, but downregulated in the mature CNS and further suppressed by axonal injury^44^,^45^. Recent studies have highlighted mTOR as a key determinant of neuronal survival and axon regeneration in response to CNS injury^46^. Conditional deletion of PTEN in adult RGC markedly promotes mTOR activation, leading to increased neurons survival and robust axons regeneration after ONI^27^,^28^. In line with these findings, we show that global inhibition of mTOR pathways by rapamycin exacerbates RGC death and subsequent neurodegeneration, although a boost in Tregs expansion can be observed after rapamycin administration (Fig. 5). Given the essential role of mTOR activity in determining the neuronal survival and axon regeneration, our data herein indicate that manipulations of mTOR signaling remain cautious to precisely target the specific cell types to assure their therapeutic effects in neural injury.

In conclusion, our study reveals that neural antigen-tolerance triggers the protective immunity of Tregs against detrimental neuroinflammation after ONI. Adoptive transfer of immunized Tregs with mTOR inhibition significantly ameliorates the progression of acute retinal injury. This study also highlights the dynamic bioactivities of mTOR signaling in the neuroimmune system. Therapeutic strategies of targeting-mTOR should be taken with specific caution that mTOR modification in different cell types may result in reverse outcomes of neuron injury. A better understanding on the interaction between nerve and immune system remains necessary for future clinical therapies that primarily target the Tregs compartment.

## Methods

### Animals

Male C57BL/6 mice aged 8–10 weeks weighing 20–22 g were used for all animal experiments. Animals were obtained from the animal facility in the Second Xiangya Hospital and maintained under specific pathogen-free conditions. All animal experiments were performed in full compliance with the ARVO Statement for the Use of Animals in Ophthalmic and Vision Research and the National Institute of Health Guide for the Care and Use of Laboratory Animals. All experimental protocols were approved by the Animal Care and Use Committee at Xiangya Medical School of Central South University.

### Induction of acute optic nerve ischemia

ONI was induced as previously described^47^. Briefly, adult mice were anesthetized by intraperitoneal injection of a ketamine (120 mg/kg)/xylazine (12 mg/kg) mixture. The anterior chamber of an eye was cannulated with a 30-gauge needle connected to a saline reservoir. For the experimental groups, the intraocular pressure (IOP) was increased to 120 mmHg in the right eyes by raising the bottle height and maintained at this pressure for 60 minutes. The left eyes of the animals served as the control were cannulated for the same amount of time without opening the outlet of the saline reservoir. Body temperatures were monitored and maintained at 37 °C using a temperature-controlled heating pad during the surgery.

### MBP inoculation

To immunize mice before ONI, bovine MBP (25 μg/5 μl; Sigma Aldrich), OVA (25 μg/5 μl; Sigma Aldrich) or normal saline (5 μl; vehicle) was instilled into each nostril every other day for over 2 weeks (a total of 7 treatments), as similarly described by previous studies^4^,^48^.

### Immunofluorescent staining

All stainings of retina samples were performed on 10 μm cryosections or whole retinas (retinal flat mount) as previously described^49^. Transverse sections or retinal flat mounts were incubated with a primary antibody against β-III-tubulin (Tuj-1; Sigma-Aldrich) or CD11b (Abcam). Related isotype immunoglobulins (Jackson ImmunoResearch) were used as negative controls in all stainings. The loss of retinal ganglion cell (RGC) was assessed in retinal flat mount as previously described^49^,^50^. All immunofluorescent assays were repeated at least three times and representative images were presented.

### Transmission electron microscopy (TEM)

TEM assays were performed for quantification of RGC axonal loss and demyelination after ONI as previously described^49^. Optic nerves were dissected and fixed in Karnovsky solution (50% in phosphate buffer) overnight. Semithin cross-sections of the nerve taken at 1.5 mm posterior to the globe were stained with toluidine blue to reveal the nerve structure. Ultrathin (60–90 nm) optic nerve cross-sections were then prepared and stained with uranyl acetate and lead citrate and were examined with a transmission electron microscope (H7500, Hitachi). Ten standard rectangular regions (234 μm^2^) randomly selected from each optic nerve section were photographed at 3000x magnification. All axons in the photomicrograph were counted, and axonal densities were calculated by averaging the data obtained from ten regions. The percentage of RGC axonal loss and demyelinated axons was calculated by dividing the number of axons in nerve sections of the surgery eyes with that of the contralateral eyes.

### Preparation of single cell suspensions

After perfusion with ice-cold PBS, mice retinas were finely minced into 1 to 2 mm^3^ pieces and digested with 1 mg/ml collagenase D (Sigma-Aldrich) and 100 μg/ml DNase I (Roche) in HBSS for 30 minutes at 37 °C. A fine cell suspension was obtained by gently pipetting the tissues up and down. The digested cell suspensions were then passed through a 40-μm cell strainer. Mice spleens and lymph nodes were mashed and filtered through the cell strainer.

### Cells isolation and functional assay

Single cell suspensions were prepared from mice spleens in RPMI-1640 media (Sigma) containing 10% FBS, 1% penicillin/streptomycin, and 1% L-glutamine. CD4+ T cells were purified using an auto MACS Separator and a CD4+ T Cell Isolation Kit (Miltenyi Biotec) according to the manufacturer’s protocol. The procedure yielded purity higher than 90% CD4+ T cells, as assessed by flow cytometry. Naïve CD4+ T cells (1 × 10^5^) were then treated with SF1670 (500 nM, Sigma), ly294002 (10 μM, Cell signaling) or rapamycin (25 nM, LC labs) for 1 hour, followed by stimulation with CD3/CD28 MACSiBead particles (at a bead-to-cell ratio of 3:1) for 36 hours (Miltenyi Biotec). Cells culture supernatants were then collected and applied to ELISA assays of IL-10 and TGF-β1 (eBioscience) according to the manufacturer’s protocol.

### Adoptive transfer

CD4+CD25+ Tregs were isolated from mice spleens using magnetic beads separation kit (Miltenyi Biotec). Briefly, the non-CD4+ T cells in cell suspensions were firstly removed using a cocktail of lineage specific biotin-conjugated antibodies against CD8, CD11b, CD45R, CD49b, Ter-119, and anti-biotin micro-beads. After that, CD25+ PE-labeled cells were magnetically retained from the purified CD4+ fractions, while the unlabeled CD4+CD25− cells ran through the column and were collected for further assays as responder T cells. The purity of CD4+CD25+ T cells routinely reached 90–95% in this study as confirmed by flow cytometry analysis. After isolation, Tregs were cultured for 1 h with the presence or absence of rapamycin (25 nM), followed by intravenous injection of 2 × 10^6^ cells via mouse-tail vein upon ONI.

### Flow cytometry assay

For intracellular staining of Foxp3, ki67, and cytokines, single cell suspensions were fixed and permeabilized using the Foxp3/Transcription factor staining buffer set (eBioscience) according to the manufacturer’s protocol. Cells were stained for 30 minutes at 4 °C with the following directly conjugated antibodies: CD3-PE/CY7, CD4-PerCP/Cy5.5, Foxp3-PE (BioLegend), ki67-AlexaFluor488, IFNγ-AlexaFluor488 (BD Biosciences), IL10-AlexaFluor488 (eBioscience), and Phospho-S6-AlexaFluor488 (Cell Signaling Technology). In addition, anti-mouse CD11b-AlexaFluor488 (BioLegend) was applied to the assessment of retinal macrophages. Acquisition and analysis of cells sorting data were performed on BD FACSCanto II using FlowJo software (Tree Star).

### ELISPOT assay

The frequency of IFNγ–producing cells was determined by mouse IFN-γ ELISPOT assay (eBioscience). After isolation, mouse splenocytes (1 × 10^5^ cells/well) were added into 96-well plates and cultured in media with MBP (25 μg/mL) for 48 hours before the next examinations. The positive spots were counted under a dissecting microscope. Results were shown as the mean number of positive spots after background subtraction from the control wells.

### Real-time PCR analysis

Real-time PCR was performed as previously described to determine the genes expressions in isolated cells or retinal tissues^51^. In brief, total RNA was extracted from cell or retinal lysates using an RNeasy Micro Kit (Qiagen). cDNA was synthesized using a Synthesis Kit (Bio-rad). Total cDNA (1 μl) was loaded in each well and mixed with PCR master mix (Applied Biosystems) and pre-designed primers (IDT, San Diego) as listed in Table 1. Gene expressions (evaluated as fold changes for each target gene) were normalized to glyceraldehyde-3-phosphate dehydrogenase (GAPDH, a housekeeping gene) following the delta-delta method. All assays were performed in triplicate. In addition, a non-template control was included in the experiment to estimate DNA contamination of isolated RNA and reagents.

### Western blot assay

Lysates of cell or tissue samples were prepared as previously described^51^. Membranes were incubated with the following primary antibodies: IL-2Rβ, P-JAK, JAK (Santa Cruz), STAT5, P-STAT5 (BioLegend), AKT, P-AKT, mTOR, P-mTOR, S6, P-S6, S6K, P-S6K (Cell Signaling Technology), Foxp3, and β-actin (Abcam). Horseradish peroxidase–conjugated secondary antibodies were applied and enhanced chemiluminescence (Thermo) was used to visualize bands.

### Statistical analysis

Statistical analysis was performed using the SPSS 22.0 software package. Results were expressed as mean ± SD. Comparisons of two groups were made using Student’s t-test. One-way ANOVA was performed for comparisons of three or more groups followed up with Tukey’s test. A P-value ≤ 0.05 was considered statistically significant.

## Additional Information

**How to cite this article**: Chen, G. *et al*. mTOR regulates neuroprotective effect of immunized CD4+Foxp3+ T cells in optic nerve ischemia. *Sci. Rep.* **6**, 37805; doi: 10.1038/srep37805 (2016).

**Publisher's note:** Springer Nature remains neutral with regard to jurisdictional claims in published maps and institutional affiliations.

## Figures and Tables

**Table 1:** Applied Primers for Real-time qPCR.

Genes	GenBank accession	Sense primers (5′-3′)	Anti-sense primers (5′-3′)
IL-10	NM_010548	GCTGGACAACATACTGCTAACC	ATTTCCGATAAGGCTTGGCAA
IL-1β	NM_008361	GAAATGCCACCTTTTGACAGTG	CTGGATGCTCTCATCAGGACA
MCP-1	NM_011333	TAAAAACCTGGATCGGAACCAAA	GCATTAGCTTCAGATTTACGGGT
TNF-α	NM_013693	CAGGCGGTGCCTATGTCTC	CGATCACCCCGAAGTTCAGTAG
TGF-β_1_	NM_011577	CCACCTGCAAGACCATCGAC	CTGGCGAGCCTTAGTTTGGAC
IFN-γ	NM_008337	ATGAACGCTACACACTGCATC	CCATCCTTTTGCCAGTTCCTC
GAPDH	NM_008084	AGGTCGGTGTGAACGGATTTG	GGGGTCGTTGATGGCAACA

Abbreviations: IL-10 - interleukin 10; IL-1β - interleukin 1 beta; MCP-1 - Monocyte Chemoattractant Protein-1; TNF-α - tumor necrosis factor alpha; TGF-β1 - transforming growth factor beta 1; IFN-γ - interferon gamma; GAPDH - glyceraldehyde-3-phosphate dehydrogenase.
